# The Two-Cent Newspaper

**Published:** 1917-01

**Authors:** 


### The Two-Cent Newspaper.

The penny papers have generally increased their price to two cents, owing to the greatly increased cost of paper. This factor does not apply so forcibly to journals of moderate issue, for which the cost of type-setting, etc., are relatively more important. It should be remembered, however, that there has been a steady increase in the cost of the mechanic part of publication in the past few years, and we, like others, may be forced to reduce the number of pages or to increase subscription rates. The latter alternative is contrary to the policy of magazines generally. We mention as a fact and not as a complaint, that the manipulator of the keyboard of a linotype machine usually receives a considerably higher rate of compensation than the one who composes on a similar keyboard, matter for publication. We do not refer to professional typewriters but to editors, reporters, and authors, excepting those of established reputation contributing to literary magazines. This exception does not apply in general to authors of scientific and professional books and magazines. In spite of the fact that a newspaper or literary magazine printing many thousand copies of an issue really feels the increased cost of paper to a considerable degree, we venture the opinion that the real reason for the increase in price of newspapers is not the higher cost of paper, but the difficulty of securing sufficient advertising. And the chief obstacle is the competition of circular, bill board and other forms of publicity. The ultimate consumer of newspapers, magazines and advertised commodities, should remember this.

